# Reply: No grey matter alterations in longitudinal data of migraine patients

**DOI:** 10.1093/brain/awaa301

**Published:** 2020-11-11

**Authors:** Matthew J Burke, Michael D Fox

**Affiliations:** a1 Harquail Centre for Neuromodulation and Hurvitz Brain Sciences Program, Sunnybrook Research Institute, Toronto, ON, Canada; a2 Neuropsychiatry Program, Department of Psychiatry, Sunnybrook Health Sciences Centre, University of Toronto, Toronto, ON, Canada; a3 Berenson-Allen Center for Noninvasive Brain Stimulation, Department of Neurology, Beth Israel Deaconess Medical Center, Harvard Medical School, Boston, MA, USA; a4 Center for Brain Circuit Therapeutics, Departments of Neurology, Psychiatry, and Radiology, Brigham and Women’s Hospital, Harvard Medical School, Boston, MA, USA; a5 Athinoula A. Martinos Centre for Biomedical Imaging, Departments of Neurology and Radiology, Massachusetts General Hospital, Harvard Medical School, Charlestown, MA, USA

We thank Mehnert and colleagues  (2020) for their interest in our article (Burke *et al.*, 2020). The authors report that they found no longitudinal grey matter changes in a sample of seven migraine patients over a 30-day period. Combined with a previous letter by Sheng *et al.* (2020), they conclude that ‘there is no robust evidence that migraine patients have structural brain changes’ and prior reports of such changes may be ‘epiphenomena’. Because we used coordinates of structural brain changes as input into our network mapping analysis, they suggest that our network findings may reflect ‘false-positives.’

We agree that it remains unclear whether structural brain changes exist in migraine, under what conditions, and whether such changes are a cause, consequence, or epiphenomenon. As noted by both Mehnert *et al*. (2020) and Sheng *et al.* (2020), some studies have reported structural differences in migraine while others have not. Depending on the meta-analysis, there may be no consistent findings across studies (Sheng *et al.*, 2020) or consistency that implicates a variety of different brain regions (Jia and Yu, 2017). This heterogeneity in neuroimaging findings is not unique to migraine, but an issue for neuroimaging studies in general (Darby *et al.*, 2018*b*).

The goal of our study was to test whether network mapping could help make sense of this heterogeneity, not to determine whether structural neuroimaging abnormalities ‘exist’ in migraine. As such, we used the most recently published meta-analysis of structural changes in migraine (Jia and Yu, 2017). Because this meta-analysis reported coordinates of structural changes, we used those coordinates as input into our network analysis. If no consistent changes had been reported (as in the meta-analysis by Sheng *et al.*, 2020), we would have performed network-mapping at the individual study level (Darby *et al.*, 2018*a*, *b*; Weil *et al.*, 2019). If no consistent changes had been reported in any of the individual studies (as in the study by Mehnert *et al*., 2020) we could have performed network mapping at the individual subject level, using single-subject patterns of brain atrophy (Tetreault *et al.*, 2020). However, it is worth noting that the 30-day time interval used in Mehnert *et al.* may not be sufficient to detect longitudinal changes in grey matter volume, even at the single-subject level (Obermann *et al.*, 2009; Rodriguez-Raecke *et al.*, 2009; May, 2011).

We disagree with the suggestion of Mehnert *et al.* that the network mapping results in Burke *et al.* represent ‘false-positives’. Rather, we accurately show that the heterogenous neuroimaging coordinates reported by Jia and Yu (2017) map to a common brain network. We welcome future work applying this network mapping approach to heterogenous findings across individual neuroimaging studies in migraine (Darby *et al.*, 2018*a*, *b*; Weil *et al.*, 2019), or heterogeneous findings across individual migraine patients (Tetreault *et al*., 2020). These different network mapping approaches appear to converge on a common brain network in Alzheimer’s disease (Darby *et al.*, 2018*b*; Ferguson *et al.*, 2019; Tetreault *et al*., 2020), and it would be interesting to see if they converge on a common network in migraine.

Finally, Mehnert *et al.* suggest using coordinates from functional neuroimaging studies rather than structural neuroimaging studies as inputs for network mapping of migraine. This is a reasonable suggestion but is likely to be more complicated than network mapping of structural changes given the wide methodological heterogeneity of functional neuroimaging studies of migraine. This includes variability in data collection (e.g. different modalities, scanning states, tasks, timing during the migraine cycle, provocative stimuli for inducing migraine etc.), and analysis techniques (e.g. different preprocessing protocols, region of interest analyses etc.). Such issues have impeded the ability to conduct appropriate functional neuroimaging meta-analyses of migraine, and accordingly systematic reviews of this literature have largely been qualitative (Schwedt *et al.*, 2015). Nevertheless, network mapping could be an ideal technique for linking heterogenous functional neuroimaging findings in migraine to a common brain network, and we encourage such efforts.

## Data availability

Data sharing is not applicable to this article as no new data were created or analysed in this study.

## Competing interests

M.J.B. has nothing to disclose. M.D.F. has intellectual property on using connectivity imaging to guide brain stimulation but receives no royalties.
